# Muscle Loss During Androgen Deprivation Therapy Is Associated With Higher Risk of Non-Cancer Mortality in High-Risk Prostate Cancer

**DOI:** 10.3389/fonc.2021.722652

**Published:** 2021-09-17

**Authors:** Pai-Kai Chiang, Wei-Kung Tsai, Allen Wen-Hsiang Chiu, Jhen-Bin Lin, Feng-Yi Yang, Jie Lee

**Affiliations:** ^1^Department of Biomedical Imaging and Radiological Sciences, National Yang Ming Chiao Tung University, Taipei, Taiwan; ^2^Department of Urology, MacKay Memorial Hospital, Taipei, Taiwan; ^3^Department of Medicine, MacKay Medical College, New Taipei City, Taiwan; ^4^MacKay Junior College of Medicine, Nursing, and Management, Taipei, Taiwan; ^5^PhD Program in Nutrition and Food Science, Graduate Institute of Biomedical and Pharmaceutical Science, Fu-Jen Catholic University, New Taipei City, Taiwan; ^6^Department of Radiation Oncology, Changhua Christian Hospital, Changhua, Taiwan; ^7^Biophotonics and Molecular Imaging Research Center, National Yang Ming Chiao Tung University, Taipei, Taiwan; ^8^Department of Radiation Oncology, MacKay Memorial Hospital, Taipei, Taiwan

**Keywords:** prostate cancer, androgen deprivation therapy, sarcopenia, body composition, non-cancer mortality, skeletal muscle loss

## Abstract

The changes in body composition are early adverse effects of androgen deprivation therapy (ADT); however, their prognostic impact remains unclear in prostate cancer. This study aimed to evaluate the association between body composition changes and survival in patients with high-risk prostate cancer. We measured the skeletal muscle index (SMI) and total adipose tissue index (TATI) at the L3 vertebral level using computed tomography at baseline and within one year after initiating ADT in 125 patients with high-risk prostate cancer treated with radiotherapy and ADT between 2008 and 2018. Non-cancer mortality predictors were identified using Cox regression models. The median follow-up was 49 months. Patients experienced an average SMI loss of 5.5% over 180 days (95% confidence interval: -7.0 to -4.0; *p*<0.001) and TATI gain of 12.6% over 180 days (95% confidence interval: 9.0 to 16.2; *p*<0.001). Body mass index changes were highly and weakly correlated with changes in TATI and SMI, respectively (Spearman ρ for TATI, 0.78, *p*<0.001; ρ for SMI, 0.27, *p*=0.003). As a continuous variable, each 1% decrease in SMI was independently associated with a 9% increase in the risk of non-cancer mortality (hazard ratio: 1.09; *p*=0.007). Moreover, the risk of non-cancer mortality increased 5.6-fold in patients with SMI loss ≥5% compared to those with unchanged SMI (hazard ratio: 5.60; *p*=0.03). Body mass index and TATI were not associated with non-cancer mortality. Muscle loss during ADT is occult, independent of weight change, and independently associated with increased non-cancer mortality in patients with high-risk prostate cancer.

## Introduction

The National Comprehensive Cancer Network (NCCN) recommendation for the treatment of patients with high-risk prostate cancer is external-beam radiotherapy (EBRT) and long-term androgen deprivation therapy (ADT) (1–4). Long-term ADT improves disease-free or overall survival in patients with high-risk prostate cancer. As a result, consideration of treatment-related morbidity has become increasingly important (3).

During ADT, most patients experience metabolic changes, such as an increase of weight and fat mass, and decreased muscle mass (3–10). The changes in body composition are early adverse effects of the treatment and can be significant within the first 3–6 months of therapy (3). In the human body, skeletal muscle and adipose tissue act as endocrine organs that secret cytokines and bioactive peptides (e.g., myokines and adipokines), which can affect whole body metabolism and inflammation (11). Progressive decrease in muscle and increase in fat increase the risk of diabetes, cardiovascular disease, falls, fractures, impaired physical activity, and disabilities (6–8). However, the effect of body composition changes on survival outcomes during ADT remains unclear.

**Figure 1:**
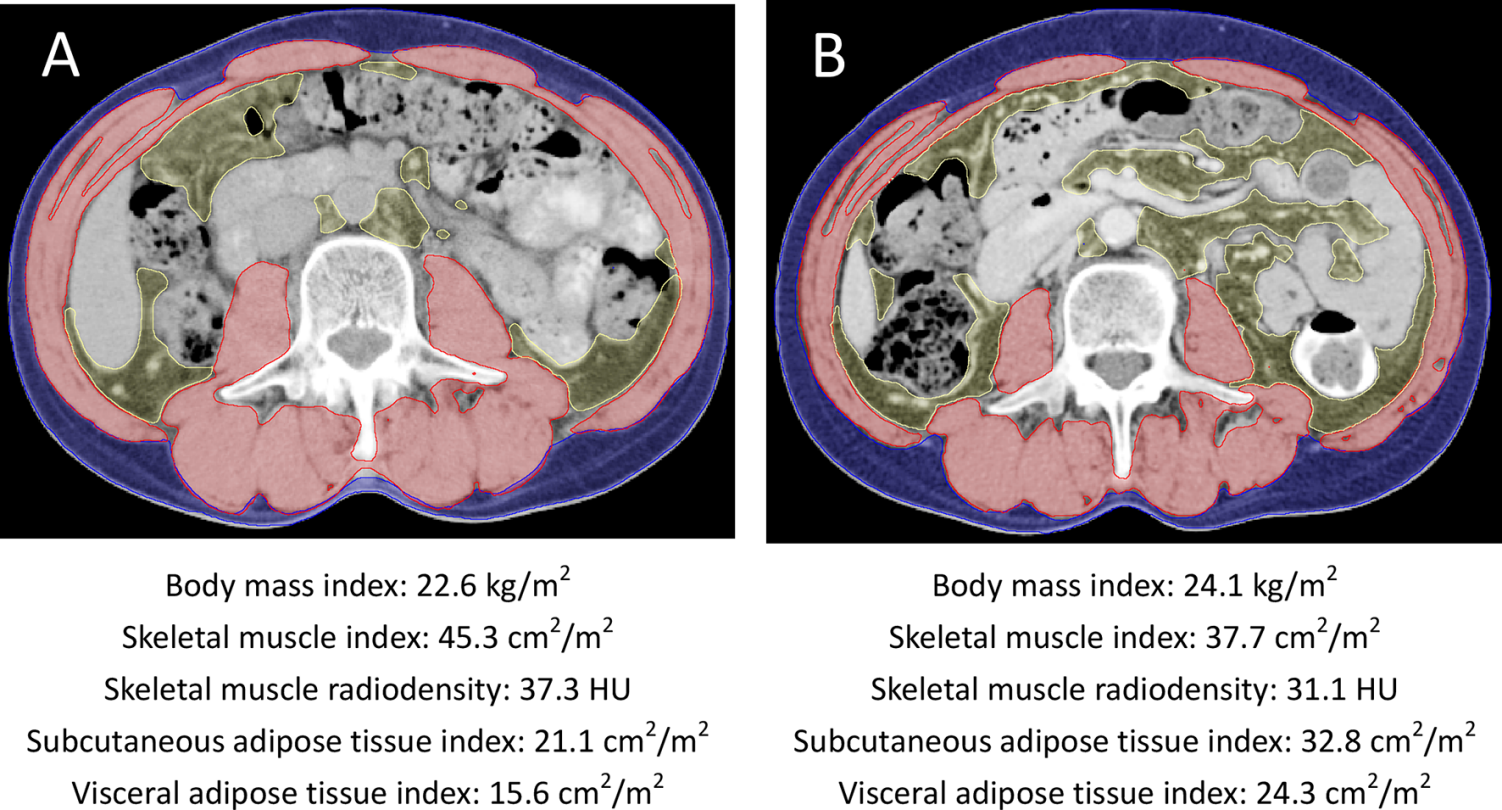
Axial cross-sectional CT images at the L3 vertebral level **(A)** at baseline and **(B)** at 6 months after initiation of radiotherapy and androgen deprivation therapy from one patient. The skeletal muscle areas, subcutaneous adipose tissue areas, and visceral adipose tissue areas were outlined in red, blue, and yellow, respectively. This patient had increased body mass index, subcutaneous and visceral adipose tissue indexes and decreased skeletal muscle index during radiotherapy and androgen deprivation therapy.

Body composition can be objectively measured by computed tomography (CT) (**Figure 1**). The cross-sectional areas of the skeletal muscle and adipose tissue on a single CT slice at the level of the third lumbar vertebrae (L3) are strongly correlated with the total body skeletal muscle and adipose mass (12–14). The skeletal muscle radiodensity (SMD) is a surrogate measure of muscle quality, and skeletal muscle with low SMD is suggestive of fatty infiltration of the skeletal muscle and poor “quality” skeletal muscle (15). A longitudinal study of CT-based composition measures may elucidate how body composition changes impact survival outcomes in these patients (16–25).

This study aimed to assess early CT-based body composition changes, and to evaluate whether body composition changes are associated with the survival outcomes of patients with high-risk prostate cancer undergoing EBRT and long-term ADT.

## Patients, Materials, and Methods

### Patients and Treatments

Our Institutional Review Board (IRB) approved this study, with waiver of informed consent owing to the retrospective and observational nature of the study. We evaluated data of 166 patients with NCCN high or very-high risk prostate cancer undergoing EBRT and long-term ADT at our institution between 2008 and 2018. The inclusion criteria were CT scans inclusive of the L3 vertebral level at baseline and within one year after treatment initiation. Patients were excluded from the analysis if they showed one of the following criteria: history of other malignancy before and/or at the time of prostate cancer diagnosis (*n*=3), missing required clinical data (*n*=6), no second CT scans within one year after treatment initiation (*n*=28), and scans of insufficient quality (*n*=4). A total of 125 patients were included in the final analysis.

Patients received combined modality therapy comprising EBRT and ADT. EBRT included intensity-modulated radiotherapy at a dose of 72–76 Gy. The course of ADT consisted of concomitant ADT with EBRT followed by adjuvant ADT for 2–3 years. The imaging modalities for pre-treatment evaluation and follow-up were CT or magnetic resonance imaging (MRI) at the discretion of the treating physicians. Body weight and height were obtained from medical records within 2 weeks of the date of the CT scans. Body mass index (BMI) was calculated as body weight divided by height in meters square. The age-adjusted Charlson Comorbidity Index (CCI) at the time of prostate cancer diagnosis was calculated for each patient by using past medical history.

### Computed Tomography-Based Body Composition Analysis

The abdominal and pelvic CT images were acquired according to a standardized protocol. Contrast agents iohexol 300 (Omnipaque 300, GE Healthcare) or iopromide 300 (Ultravist 300, Bayer HealthCare) were intravenously administered in a single uniphasic bolus dose of 80–100 mL *via* a power injector at 2 mL/s. The portal-venous phase was obtained with a fixed delay of 70 seconds after the administration of the contrast material. The CT image parameters included the following features: contrast-enhancement, 3-mm slice thickness, 120 kVp, and approximately 290 mA.

A single slice of CT scans at L3 level was used to analyze cross-sectional areas (cm^2^) of the skeletal muscle (including the psoas, rectus abdominis, paraspinal, transversus abdominis, and internal and external oblique muscles), and visceral and subcutaneous adipose tissues by using the Varian Eclipse software (Varian Medical Systems Inc., Palo Alto, CA, USA) (**Figure 1**) (12–14). Body composition was defined based on Hounsfield unit (HU) thresholds, which ranged from −29 to +150 HU for skeletal muscle, from −50 to −150 HU for visceral adipose tissue, and from −30 to −190 HU for subcutaneous tissue. The mean radiation attenuation of the skeletal muscle was the SMD. The total adipose tissue (TAT) area was calculated as the sum of the areas of the subcutaneous and visceral adipose tissues. One researcher, blinded to the patient information, measured the body composition parameters. The cross-sectional areas of the skeletal muscle, visceral adipose tissue, subcutaneous adipose tissue, and total adipose tissue were normalized for the patient height to calculate indexes (cm^2^/m^2^) for skeletal muscle (SMI), subcutaneous adipose tissue (SATI), visceral adipose tissue (VATI), and total adipose tissue (TATI) (17).

As body composition varies greatly between regions, ethnicities, and cancer types, we defined our own cut-off values for defining sarcopenia on the basis of previous studies with similar population sizes (17–19). Cut-off values were set at the lowest tertile for SMI. The body composition change was the difference between pre-treatment and follow-up CT scans. In this study, the median duration between pre-treatment and follow-up CT scans was 180 days (interquartile range [IQR]: 146–223 days). To account for variations in the scan interval duration, body composition changes were normalized as the change over 180 days for providing a standardized unit for comparison between patients. Previous studies had reported that SMI loss ≥5% was associated with a poor survival outcome in cancer patients (20–23). In this study, patients with a reduction in SMI ≥5% were categorized as “loss”, and those with an SMI gain or reduction <5% were categorized as “maintain”.

### Statistical Analysis

Continuous data are summarized as mean ± standard deviation or median and IQR, as applicable, while categorical data are summarized as numbers and percentages. The distributions of patient characteristics were compared using the chi-square test for categorical variables and independent *t*-test or Mann-Whitney *U* test for continuous variables, as statistically appropriate. Paired *t*-tests and the Wilcoxon signed-rank test were used to assess body composition changes. The McNemar’s test was used to test for significant differences in paired categorical data. The Spearman correlation coefficient was used to assess relationships among body composition parameters.

The primary end point was death from causes other than prostate cancer. Cause of death was verified *via* death certificate. Non-cancer-specific survival was measured from the date of diagnosis of prostate cancer to the date of death from causes other than prostate cancer. Prostate cancer-specific survival was measured from the date of diagnosis of prostate cancer to the date of death from prostate cancer. Survival curves were constructed using the Kaplan-Meier method with log-rank tests. Cox proportional hazard models were used to estimate the hazard ratio (HR) and 95% confidence interval (CI). The multivariable models were selected by backward elimination with a 0.05 significance level for inclusion. The data were analyzed using IBM SPSS software (version 21.0; IBM Corp., Armonk, NY, USA). A *p*<0.05 was considered statistically significant.

## Results

The patient and tumor characteristics of the 125 patients are presented in **Table 1**. The median age was 73 years (IQR: 67–78 years), and the median age-adjusted CCI was 4 (IQR: 3–5). Ninety (72.0%) and 35 (28.0%) patients were classified as having NCCN high-risk and very high-risk diseases, respectively. The median follow-up was 49 months (IQR: 28–75 months). No detectable fractures occurred during the follow-up. Sixteen (12.8%) non-cancer deaths and 17 (13.6%) prostate cancer deaths were observed. The causes of non-cancer deaths were pneumonia (*n*=9), ischemic heart disease (*n*=5), and stroke (*n*=2).

**Table 1:** Patient and tumor characteristics.

Characteristics	Overall (*n* = 125)
**Age (years), median (IQR)**	73 (67-78)
**Age-adjusted CCI score**	
1	4 (3.2)
2	27 (21.6)
3	22 (17.6)
4	33 (26.4)
5	33 (26.4)
6	6 (4.8)
**NCCN risk groups**	
High	90 (72.0)
Very high	35 (28.0)
**PSA level (ng/mL), median (IQR)**	27.8 (21.2-60.5)
**Body composition at baseline**	
BMI (kg/m^2^)	24.2 ± 3.5
SMI (cm^2^/m^2^)	47.2 ± 7.4
Sarcopenia^a^	42 (33.6)
SMD (HU)	37.8 ± 5.6
SATI (cm^2^/m^2^)	39.9 ± 13.6
VATI (cm^2^/m^2^)	55.2 ± 28.0
TATI (cm^2^/m^2^)	95.1 ± 37.7
**Median (IQR) duration between CT scans, days**	180 (146-223)

BMI, body mass index; CCI, Charlson Comorbidity Index; CT, computed tomography; HU, Hounsfield unit; IQR, interquartile range; NCCN, National Comprehensive Cancer Network; SMD, skeletal muscle radiodensity; SMI, skeletal muscle index; SATI, subcutaneous adipose tissue index; TATI, total adipose tissue index; VATI, visceral adipose tissue index.

Data are mean ± standard error or number (%).

a SMI<43.2 cm^2^/m^2^ were defined as sarcopenia.

### Body Composition Changes During Radiotherapy and ADT

**Figure 2:**
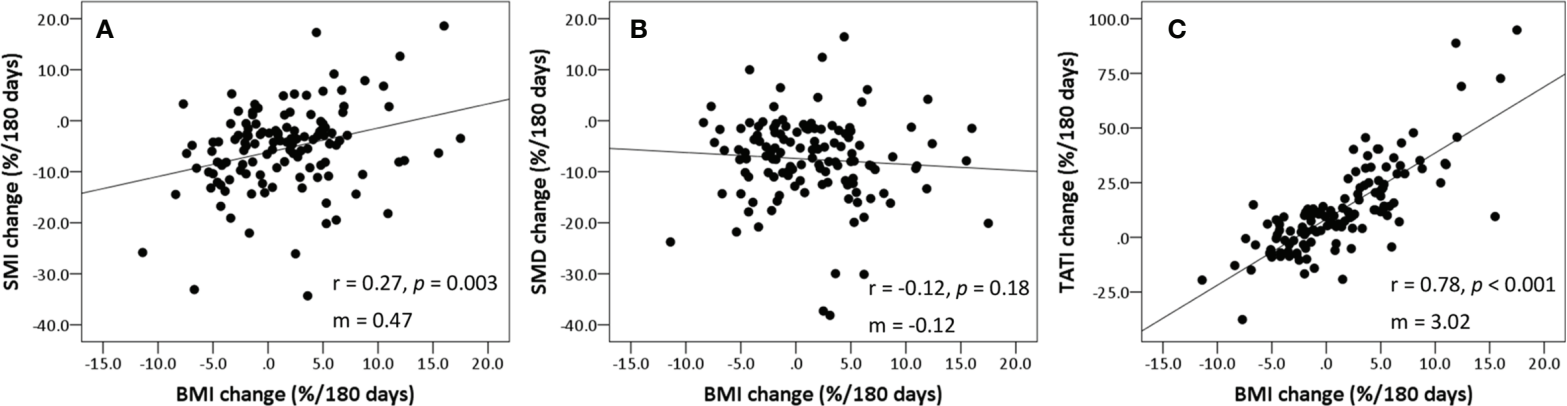
Scatter plots showing correlation between the changes in BMI, SMI, SMD, and TATI. **(A)** SMI *vs*. BMI, **(B)** SMD *vs*. BMI, and **(C)** TATI *vs*. BMI. Spearman’s rank correlation coefficient (rho) was used to assess correlation between body composition parameters. Slopes (m) for the correlations are shown on each graph. BMI, body mass index; SMD, skeletal muscle radiodensity; SMI, skeletal muscle index; TATI, total adipose tissue index.

**Table 2** summarizes the body composition characteristics at baseline and the changes during treatment. The sarcopenia cut-off value was set at SMI <43.2 cm^2^/m^2^, which corresponds to the lowest tertile. Overall, the BMI, SATI, VATI, and TATI increased, while SMI and SMD decreased during treatment. The prevalence of sarcopenia increased from 33.6% (*n*=42) at baseline to 48.0% (*n*=60) at the time of the second CT scan (*p*=0.001). Fifty-eight patients (46.4%) experienced SMI loss of ≥5%. The changes in BMI were weakly correlated to the changes in SMI and SMD (Spearman ρ for SMI, 0.27; *p*=0.003; ρ for SMD, -0.12; *p*=0.18) (**Figure 2**). The changes in BMI showed a high positive correlation with changes in TATI (Spearman ρ for TATI, 0.78; *p*<0.001).

**Table 2:** Change of body composition parameters during radiotherapy and androgen deprivation therapy.

Variable	First CT scan	Second CT scan	Absolute change per 180 days			Relative Change per 180 days (%)		
	Mean ± SD	Mean ± SD	Mean	95% CI	*p*-value	Mean	95% CI	*p*-value
**BMI (kg/m^2^)**	24.2 ± 3.5	24.5 ± 3.5	0.3	0.1 to 0.5	0.005	1.4	0.5 to 2.3	0.003
**SMI (cm^2^/m^2^)**	47.2 ± 7.4	44.5 ± 7.3	-2.7	-3.4 to -2.0	<0.001	-5.5	-7.0 to -4.0	<0.001
**SMD (HU)**	37.8 ± 5.6	34.9 ± 5.9	-2.9	-3.4 to -2.3	<0.001	-7.6	-9.0 to -6.1	<0.001
**SATI (cm^2^/m^2^)**	39.9 ± 13.6	44.7 ± 14.7	5.0	3.7 to 6.3	<0.001	14.7	10.9 to 18.5	<0.001
**VATI (cm^2^/m^2^)**	55.2 ± 28.0	60.1 ± 29.6	4.9	2.9 to 7.1	<0.001	13.3	8.5 to 18.1	<0.001
**TATI (cm^2^/m^2^)**	95.1 ± 37.7	104.6 ± 40.4	9.8	6.8 to 12.7	<0.001	12.6	9.0 to 16.2	<0.001

BMI, body mass index; CI, confidence interval; CT, computed tomography; HU, Hounsfield unit; SD, standard deviation; SMD, skeletal muscle radiodensity; SMI, skeletal muscle index; SATI, subcutaneous adipose tissue index; TATI, total adipose tissue index; VATI, visceral adipose tissue index.

Patient characteristics according to pre-treatment sarcopenia are summarized in **Supplementary Table S1**. Patients with pre-treatment sarcopenia had significantly older age and lower pre-treatment BMI, SMI, SATI, and TATI. However, the changes in body composition parameters during treatment were not different between pre-treatment sarcopenia and non-sarcopenia groups. The patient characteristics according to SMI change during treatment are presented in **Supplementary Table S2**. A higher age-adjusted CCI was associated with SMI loss ≥5% (*p*=0.01). SMI at baseline was similar between the SMI loss ≥5% and SMI maintain groups (*p*=0.48); 18 (31.0%) and 24 (35.8%) patients in the SMI loss ≥5% and SMI maintain groups, respectively, had pre-treatment sarcopenia (*p*=0.57). The SMD decreased by a greater extent in patients with SMI loss ≥5% compared to those with SMI maintain (*p*<0.001); the changes in adipose tissue indexes were not significantly different between groups.

### Body Composition and Survival Outcomes

**Figure 3:**
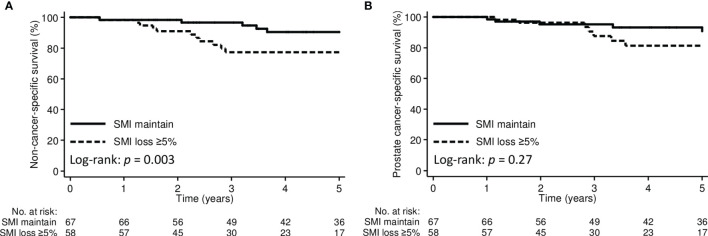
Kaplan-Meier curve demonstrating **(A)** non-cancer-specific survival and **(B)** prostate cancer-specific survival according to SMI change groups. SMI, skeletal muscle index.

The 3-year non-cancer-specific and prostate cancer-specific survival for the entire cohort were 87.9% and 92.2%, respectively. By stratifying patients according to pre-treatment sarcopenia, the 3-year non-cancer-specific survival was 84.6% in the pre-treatment sarcopenic group and 89.1% in the non-sarcopenic group (*p*=0.28); the corresponding 3-year prostate cancer-specific survival were 90.6% and 93.0%, respectively (*p*=0.91). However, the 3-year non-cancer-specific survival were significantly poorer in patients with SMI loss ≥5% than those with SMI maintain (77.3% *vs*. 96.7%, *p*=0.003) (**Figure 3**). The 3-year prostate cancer-specific survival was not different between SMI loss ≥5% and SMI maintain groups (87.5% *vs*. 95.3%, *p*=0.27).

**Table 3** shows the results of the univariable and multivariable Cox proportional hazard regression analysis for non-cancer mortality. On univariable analysis, age, age-adjusted CCI, SMI change (continuous), and SMI loss ≥5% (categorical) were associated with non-cancer mortality. On multivariable analysis, each 1% decrease in SMI over 180 days was independently associated with a 9% increase in the risk of non-cancer mortality (HR: 1.09, 95% CI: 1.02–1.16; *p*=0.007). Furthermore, as a categorical variable, an SMI loss ≥5% was associated with an increased risk of non-cancer mortality (HR: 5.60, 95% CI: 1.23–25.45; *p*=0.03). Sarcopenia at baseline, BMI, and adipose tissue indexes at baseline and changes during treatment were not associated with non-cancer mortality.

**Table 3:** Cox proportional hazards model for non-cancer mortality.

Variable	Univariable		Multivariable model 1*		Multivariable model 2*	
	HR (95% CI)	*p*-value	HR (95% CI)	*p*-value	HR (95% CI)	*p*-value
**Age (years)**	1.11 (1.02-1.21)	0.03	–	–	–	–
**Age-adjusted CCI score, continuous**	3.30 (1.66-6.55)	0.001	2.94 (1.47-5.89)	0.002	3.06 (1.52-6.15)	0.002
**NCCN risk groups**						
High	Reference					
Very high	1.77 (0.58-5.41)	0.32				
**BMI at baseline (1 kg/m^2^ increase)**	0.95 (0.81-1.10)	0.47				
**BMI change (per 1%/180 days increase)**	0.91 (0.80-1.02)	0.12				
**SMI at baseline (1 cm^2^/m^2^ decrease)**	1.04 (0.96-1.12)	0.32				
**Sarcopenia at baseline**	1.35 (0.44-4.13)	0.60				
**SMI change (per 1%/180 days decrease)**	1.09 (1.04-1.15)	0.001	1.09 (1.02-1.16)	0.007	–	–
**SMI loss ≥5% (Reference: SMI maintain)**	6.95 (1.54-31.39)	0.01	–	–	5.60 (1.23-25.45)	0.03
**SMD at baseline (1 HU decrease)**	1.08 (0.98-1.19)	0.10				
**SMD change (per 1%/180 days decrease)**	1.05 (0.99-1.11)	0.09				
**SATI at baseline (1 cm^2^/m^2^ increase)**	1.01 (0.97-1.05)	0.70				
**SATI change (per 1%/180 days increase)**	0.99 (0.96-1.02)	0.42				
**VATI at baseline (1 cm^2^/m^2^ increase)**	1.00 (0.98-1.02)	0.66				
**VATI change (per 1%/180 days increase)**	0.99 (0.97-1.02)	0.51				
**TATI at baseline (1 cm^2^/m^2^ increase)**	1.00 (0.98-1.01)	0.85				
**TATI change (per 1%/180 days increase)**	1.01 (0.98-1.04)	0.48	1.03 (0.99-1.07)	0.10	–	–

BMI, body mass index; CI, confidence interval; CCI, Charlson Comorbidity Index; CT, computed tomography; HR, hazard ratio; HU, Hounsfield unit; IQR, interquartile range; NCCN, National Comprehensive Cancer Network; SMD, skeletal muscle radiodensity; SMI, skeletal muscle index; SATI, subcutaneous adipose tissue index; TATI, total adipose tissue index; VATI, visceral adipose tissue index.

Data are mean ± standard error or number (%).

*Multivariable analysis using a backward selection method.

The results of the Cox proportional hazard regression analysis for prostate cancer-specific mortality are shown in **Supplementary Table S3**. NCCN very high-risk disease was independently associated with an increased risk of prostate cancer-specific mortality (HR: 6.28, 95% CI: 1.39–28.44; *p*=0.02). Body composition parameters at baseline and changes during treatment were not associated with prostate cancer-specific mortality.

## Discussion

This study assessed the association of CT-based body composition changes with survival outcomes in patients with high-risk prostate cancer undergoing EBRT and ADT. Body composition changed significantly after initiating ADT. More specifically, BMI and adipose tissue indexes increased, and SMI and SMD decreased. The changes in BMI were highly correlated with the changes in TATI, but weakly correlated with the changes in SMI and SMD, suggesting that weight measurement may not detect muscle loss in clinical practice. Moreover, SMI loss during ADT was independently associated with increased non-cancer mortality. BMI and adipose tissue indexes were not associated with non-cancer mortality.

The observation that early body composition changes during ADT is consistent with the results of prior studies (3, 5–7). In those studies, the lean body mass significantly decreased by 1.0% to 3.8% from baseline to 12 months after initiating ADT (5–7). However, we found a greater decrease in muscle mass of 5.5% in our patients. The possible explanation is that patients in this study may have higher age-adjusted CCI. We also found that patients with higher age-adjusted CCI were more likely to experience SMI loss during ADT.

Weight measurement may not detect muscle loss. BMI changes were more likely to be representative of fat changes in patients undergoing ADT. BMI has been previously identified as an imprecise marker of SMI, highlighting the relevance of body composition measurements (21, 26–29). However, CT scans may not be available for body composition analysis in patients with prostate cancer. This is because MRI is preferred due to its higher ability to evaluate the prostate gland. Faron et al. revealed the interchangeability of CT and MRI derived measurements of cross-sectional area and fatty infiltration of the muscle, suggesting that it may be feasible to evaluate body composition changes by MRI in patients with prostate cancer (30). MRI-based body composition measurement should be validated in further studies. Dual-energy X-ray absorptiometry, ultrasound, and bioelectrical impedance analysis are modalities that can also quantify body composition. Integration of monitoring body composition into routine cancer care should be considered for these patients.

Pre-treatment sarcopenia has been previously identified as a predictor of non-cancer mortality in prostate cancer (14). However, we found that muscle loss within one year after initiating ADT was associated with increased non-cancer mortality, while pre-treatment sarcopenia was not. Body composition changes are dynamic throughout the disease trajectory and can impact survival in patients with cancer (16–21). Notably, patients in this study received long-term ADT and we only analyzed body composition changes within one year after initiating ADT. Patients receiving long-term ADT may experience progressive muscle loss during the second and the third year of ADT (7). Future studies are needed to evaluate whether further muscle loss can affect outcomes and whether muscle can be preserved *via* multimodal interventions in these patients. Early multimodal intervention (nutrition, exercise, and anti-inflammatory medication) may maintain muscle mass after initiating ADT and improve survival in these patients (16, 31–33). Our findings need to be evaluated in future studies.

The use of ADT is associated with increased risk of falls and fracture in patients with prostate cancer (7, 34–36). Falls cause substantial morbidity and mortality and lead to a considerable burden on health systems (37). Various factors, including ageing, multiple chronic diseases, life style, malnutrition, and decline in muscle strength and physical function, determine the risks of falls (38–40). In this study, no detectable fracture occurred during the follow-up. The possible explanation of this fact might be the lack of routine spine radiographs. As vertebral fractures are mostly asymptomatic (41), asymptomatic vertebral fractures may not be detected in included patients. Furthermore, the information on falls was not available for analysis due to the retrospective study design. Therefore, the relationship between muscle loss and the risk of falls or fracture cannot be evaluated in this study. Previous studies revealed that exercises implementation can preserve muscle strength and physical function in patients with prostate cancer receiving ADT (31, 33). Therefore, multimodal interventions, including exercise and nutrition, may help preserve muscle mass and reduce the risk of falls in these patients (40, 42).

Serum testosterone is positively correlated with muscle mass in men (43). In patients with prostate cancer, the aim of ADT is to suppress serum testosterone to castration level. The suppression of testosterone may therefore contribute to the decrease in muscle mass in these patients. In addition, the lower nadir of serum testosterone may be associated with clinical outcomes including survival (44). Regular measurement of testosterone was also suggested in men receiving ADT to ensure adequate testosterone suppression (45). However, serum testosterone levels were not available in all patients included in this study, thus limiting the possibility to further elucidate the association of serum testosterone with muscle loss in these patients.

This study has some limitations. This was a retrospective investigation with a small number of patients and variable durations between CT scans. Only 125 of 166 identified patients were included in the analysis owing to missing data (no second CT image) within one year after the initiation of ADT. Information on diet, physical activity, and falls was not available for analysis due to the retrospective design of the study. The death certificates may not always provide the real cause of death. Selection bias and residual and unmeasured confounding are also potential limitations. In addition, this study only analyzed CT scans at 2 timepoints acquired during routine cancer care. Body composition measurements using more time points may provide more comprehensive information (7). It is also unknown whether improving muscle mass after early muscle loss can optimize survival outcomes. Therefore, the findings of this study should be validated in a larger sample size to determine the prognostic significance of muscle loss in patients with high-risk prostate cancer.

In summary, muscle loss occurs in patients with high-risk prostate cancer undergoing EBRT and ADT. Patients with SMI loss ≥5% at 6 months after initiating ADT have an increased risk of non-cancer mortality compared with patients in the SMI maintain group. Moreover, muscle loss is occult and independent of weight change, suggesting that body composition measurements should be incorporated into clinical practice. Future studies are required to evaluate early multimodal interventions with a goal of muscle maintenance; this would serve to optimize the survival outcomes of patients with high-risk prostate cancer.

## Publisher’s Note

All claims expressed in this article are solely those of the authors and do not necessarily represent those of their affiliated organizations, or those of the publisher, the editors and the reviewers. Any product that may be evaluated in this article, or claim that may be made by its manufacturer, is not guaranteed or endorsed by the publisher.

## Data Availability Statement

The raw data supporting the conclusions of this article will be made available by the authors, without undue reservation.

## Ethics Statement

The studies involving human participants were reviewed and approved by MacKay Memorial Hospital, Taipei, Taiwan. Written informed consent for participation was not required for this study in accordance with the national legislation and the institutional requirements.

## Author Contributions

P-KC, F-YY, and JL designed the research. P-KC and JL analysed data and wrote this manuscript. W-KT contributed in performing the research. J-BL contributed in performing the image data analysis. AW-HC revised this manuscript critically for important intellectual content. All authors contributed to the article and approved the submitted version.

## Conflict of Interest

The authors declare that the research was conducted in the absence of any commercial or financial relationships that could be construed as a potential conflict of interest.

## Publisher’s Note

All claims expressed in this article are solely those of the authors and do not necessarily represent those of their affiliated organizations, or those of the publisher, the editors and the reviewers. Any product that may be evaluated in this article, or claim that may be made by its manufacturer, is not guaranteed or endorsed by the publisher.
