# Supplementary material for: Muscle Loss During Androgen Deprivation Therapy Is Associated With Higher Risk of Non-Cancer Mortality in High-Risk Prostate Cancer
Source: Front Oncol. 2021 Sep 17;11:722652. doi: 10.3389/fonc.2021.722652 (PMC8485032; doi:10.3389/fonc.2021.722652)
Supplement: Supplementary file 1 [file Table_1.docx]

**Supplementary Table S1** Patient and tumor characteristics according to pre-treatment sarcopenia groups.

| **Characteristics** | **Non-Sarcopenia^a^ (*n*=83)** | **Sarcopenia^a^ (*n*=42)** | ***p*-value** |
| --- | --- | --- | --- |
| **Age (years), median (IQR)** | 71 (66-77) | 75 (69-79) | **0.04** |
| **Age-adjusted CCI score** |  |  | 0.75 |
| 1 | 3 (3.6) | 1 (2.4) |  |
| 2 | 19 (22.9) | 8 (19.0) |  |
| 3 | 16 (19.3) | 6 (14.3) |  |
| 4 | 19 (22.9) | 14 (33.3) |  |
| 5 | 23 (27.7) | 10 (23.8) |  |
| 6 | 3 (3.6) | 3 (7.1) |  |
| **NCCN risk groups** |  |  | 0.75 |
| High | 59 (71.1) | 31 (73.8) |  |
| Very high | 24 (28.9) | 11 (26.2) |  |
| **PSA level (ng/mL), median (IQR)** | 27.8 (21.2-60.0) | 27.4 (21.0-62.1) | 0.62 |
| **BMI at baseline (kg/m^2^)** | 25.3 ± 3.2 | 22.0 ± 3.1 | **<0.001** |
| **BMI change (%/180 days)** | 1.6 ± 5.3 | 0.9 ± 5.3 | 0.45 |
| **SMI at baseline (cm^2^/m^2^)** | 51.2 ± 5.5 | 39.4 ± 3.1 | **<0.001** |
| **SMI change (%/180 days)** | -6.0 ± 8.0 | -4.6 ± 9.0 | 0.39 |
| **SMD at baseline (HU)** | 38.0 ± 5.6 | 37.5 ± 5.5 | 0.66 |
| **SMD change (%/180 days)** | -7.9 ± 8.8 | -6.8 ± 7.4 | 0.47 |
| **SATI at baseline (cm^2^/m^2^)** | 41.9 ± 12.5 | 35.9 ± 14.9 | **0.02** |
| **SATI change (%/180 days)** | 15.2 ± 21.7 | 13.6 ± 21.0 | 0.71 |
| **VATI at baseline (cm^2^/m^2^)** | 58.2 ± 27.5 | 49.3 ± 28.3 | 0.09 |
| **VATI change (%/180 days)** | 14.4 ± 25.5 | 11.2 ± 30.1 | 0.53 |
| **TATI at baseline (cm^2^/m^2^)** | 100.1 ± 35.7 | 85.2 ± 40.2 | **0.04** |
| **TATI change (%/180 days)** | 13.5 ± 20.4 | 10.8 ± 19.8 | 0.49 |

Abbreviations: BMI, body mass index; CCI, Charlson Comorbidity Index; CT, computed tomography; HU, Hounsfield unit; IQR, interquartile range; NCCN, National Comprehensive Cancer Network; SMD, skeletal muscle radiodensity; SMI, skeletal muscle index; SATI, subcutaneous adipose tissue index; TATI, total adipose tissue index; VATI, visceral adipose tissue index.

Data are mean ± standard error or number (%).

Bolded *p*-values are those significant with a *p*<0.05.

^a^ SMI<43.2 cm^2^/m^2^ were defined as sarcopenia.

**Supplementary Table S2** Patient and tumor characteristics according to SMI change groups.

| **Characteristics** | **SMI loss ≥5% (*n*=58)** | **SMI maintain (*n*=67)** | ***p*-value** |
| --- | --- | --- | --- |
| **Age (years), median (IQR)** | 74 (67-78) | 71 (66-78) | 0.22 |
| **Age-adjusted CCI score** |  |  | **0.01** |
| 1 | 1 (1.7) | 3 (4.5) |  |
| 2 | 10 (17.2) | 17 (25.4) |  |
| 3 | 14 (24.1) | 8 (11.9) |  |
| 4 | 8 (13.8) | 25 (37.3) |  |
| 5 | 21 (36.2) | 12 (17.9) |  |
| 6 | 4 (6.9) | 2 (3.0) |  |
| **NCCN risk groups** |  |  | 0.92 |
| High | 42 (72.4) | 48 (71.6) |  |
| Very high | 16 (27.6) | 19 (28.4) |  |
| **PSA level (ng/mL), median (IQR)** | 27.1 (20.4-60.3) | 28.0 (21.5-64.3) | 0.57 |
| **BMI at baseline (kg/m^2^)** | 24.0 ± 3.3 | 24.4 ± 3.7 | 0.55 |
| **BMI change (%/180 days)** | 0.3 ± 5.5 | 2.3 ± 4.9 | **0.03** |
| **SMI at baseline (cm^2^/m^2^)** | 47.7 ± 7.6 | 46.8 ± 7.2 | 0.48 |
| **Sarcopenia^a^ at baseline** | 18 (31.0) | 24 (35.8) | 0.57 |
| **Sarcopenia^a^ at second CT scan** | 36 (62.1) | 24 (35.8) | **0.003** |
| **SMD at baseline (HU)** | 37.1 ± 5.5 | 38.5 ± 5.6 | 0.17 |
| **SMD change (%/180 days)** | -11.5 ± 8.6 | -4.2 ± 6.5 | **<0.001** |
| **SATI at baseline (cm^2^/m^2^)** | 38.9 ± 11.7 | 40.8 ± 15.0 | 0.43 |
| **SATI change (%/180 days)** | 15.6 ± 23.3 | 13.9 ± 19.7 | 0.66 |
| **VATI at baseline (cm^2^/m^2^)** | 55.4 ± 25.2 | 55.0 ± 30.3 | 0.94 |
| **VATI change (%/180 days)** | 14.3 ± 24.6 | 12.5 ± 29.1 | 0.55 |
| **TATI at baseline (cm^2^/m^2^)** | 94.2 ± 33.4 | 95.8 ± 41.4 | 0.82 |
| **TATI change (%/180 days)** | 13.3 ± 20.0 | 12.0 ± 20.4 | 0.46 |
| **Median (IQR) duration between CT scans, days** | 178 (159-224) | 186 (164-226) | 0.59 |

Abbreviations: BMI, body mass index; CCI, Charlson Comorbidity Index; CT, computed tomography; HU, Hounsfield unit; IQR, interquartile range; NCCN, National Comprehensive Cancer Network; SMD, skeletal muscle radiodensity; SMI, skeletal muscle index; SATI, subcutaneous adipose tissue index; TATI, total adipose tissue index; VATI, visceral adipose tissue index.

Data are mean ± standard error or number (%).

Bolded *p*-values are those significant with a *p*<0.05.

^a^ SMI<43.2 cm^2^/m^2^ were defined as sarcopenia.

**Supplementary Table S3** Cox proportional hazards model for prostate cancer-specific mortality.

|  | **Univariable** |  |  | **Multivariable model 1*** |  |  | **Multivariable model 2*** |  |
| --- | --- | --- | --- | --- | --- | --- | --- | --- |
| **Variable** | **Hazard ratio (95% CI)** | ***p*-value** |  | **Hazard ratio (95% CI)** | ***p*-value** |  | **Hazard ratio (95% CI)** | ***p*-value** |
| **Age (years)** | 0.98 (0.89-1.08) | 0.68 |  |  |  |  |  |  |
| **Age adjusted CCI score, continuous** | 0.93 (0.54-1.62) | 0.81 |  |  |  |  |  |  |
| **NCCN risk groups** |  |  |  |  |  |  |  |  |
| High | Reference |  |  | Reference |  |  | Reference |  |
| Very high | 4.71 (1.13-19.71) | **0.03** |  | 6.28 (1.39-28.44) | **0.02** |  | 4.71 (1.13-19.71) | **0.03** |
| **BMI at baseline (1 kg/m^2^ increase)** | 1.02 (0.84-1.25) | 0.83 |  |  |  |  |  |  |
| **BMI change (per 1%/180 days increase)** | 1.09 (0.96-1.23) | 0.19 |  |  |  |  |  |  |
| **SMI at baseline (1 cm^2^/m^2^ decrease)** | 1.02 (0.93-1.12) | 0.73 |  |  |  |  |  |  |
| **Sarcopenia at baseline** | 1.25 (0.30-5.27) | 0.75 |  |  |  |  |  |  |
| **SMI change (per 1%/180 days decrease)** | 1.05 (0.97-1.13) | 0.20 |  | 1.08 (0.99-1.17) | 0.08 |  | - | - |
| **SMI loss ≥5% (Reference: SMI maintain)** | 2.19 (0.52-9.16) | 0.29 |  |  |  |  |  |  |
| **SMD at baseline (1 HU decrease)** | 0.96 (0.84-1.09) | 0.49 |  |  |  |  |  |  |
| **SMD change (per 1%/180 days decrease)** | 1.06 (0.98-1.14) | 0.14 |  |  |  |  |  |  |
| **SATI at baseline (1 cm^2^/m^2^ increase)** | 0.97 (0.92-1.03) | 0.33 |  |  |  |  |  |  |
| **SATI change (per 1%/180 days increase)** | 1.02 (0.99-1.05) | 0.17 |  |  |  |  |  |  |
| **VATI at baseline (1 cm^2^/m^2^ increase)** | 1.00 (0.97-1.02) | 0.71 |  |  |  |  |  |  |
| **VATI change (per 1%/180 days increase)** | 1.02 (0.99-1.04) | 0.14 |  |  |  |  |  |  |
| **TATI at baseline (1 cm^2^/m^2^ increase)** | 0.99 (0.98-1.01) | 0.54 |  |  |  |  |  |  |
| **TATI change (per 1%/180 days increase)** | 1.01 (0.98-1.04) | 0.42 |  |  |  |  |  |  |

Abbreviations: BMI, body mass index; CCI, Charlson Comorbidity Index; CT, computed tomography; HU, Hounsfield unit; IQR, interquartile range; NCCN, National Comprehensive Cancer Network; SMD, skeletal muscle radiodensity; SMI, skeletal muscle index; SATI, subcutaneous adipose tissue index; TATI, total adipose tissue index; VATI, visceral adipose tissue index.

Data are mean ± standard error or number (%).

Bolded *p*-values are those significant with a *p*<0.05.

* Multivariable analysis using a backward selection method.
